# Arginine–Silicate–Inositol Promotes Femoral Fracture Healing in Rats

**DOI:** 10.1007/s12011-026-05085-7

**Published:** 2026-04-06

**Authors:** Sukru Demir, Kazim Sahin, Ibrahim Hanifi Ozercan, Gokhan Once, Sefa Key, Murat Gurger

**Affiliations:** 1 Department of Orthopedics and Traumatology, Firat University Hospital, Elazig, Turkey; 2 Faculty of Veterinary Science, Department of Animal Nutrition, Firat University, Elazig, Turkey; 3 Department of Pathology, Firat University Hospital, Elazig, Turkey; 4 Clinics of Orthopedics and Traumatology, Elazig Fethi Sekin City Hospital, Elazig, Turkey

**Keywords:** Arginine-silicate-inositol complex, Fracture healing

## Abstract

The arginine-silicate-inositol (ASI) complex (Arg 49.47%, silicon 8.2%, inositol 25%) is a bioavailable source of silicon and arginine with potential benefits for bone metabolism. This study aimed to investigate the effects of oral ASI supplementation on fracture healing in a rat femoral fracture model. Twenty-eight female Wistar-Albino rats were subjected to standardized femoral osteotomy using a multiple drilling technique under anesthesia. Group 1 and Group 2 received a standard diet for 8 weeks, Group 3 received low-dose ASI, and Group 4 received high-dose ASI. Histopathological analysis demonstrated a significant increase in osteoblast numbers in ASI-treated groups, with the highest values observed in the high-dose ASI group (*p* < 0.001). Osteoclast numbers were significantly reduced in the high-dose ASI group compared with controls (*p* = 0.017). Radiological evaluation revealed the absence of union in fracture controls, whereas low-dose ASI promoted fracture union with normal callus formation, whereas high-dose ASI resulted in more extensive and mature callus formation. In conclusion, ASI supplementation significantly enhanced fracture healing, callus maturation, and new bone formation in a dose-dependent manner. These findings suggest that ASI may be a promising nutritional strategy for supporting bone repair.

## Introduction

The extended human lifespan has caused an increase in the elderly population and, therefore, osteoporosis and fractures [1]. In this respect, fracture healing and associated factors become important. Fracture healing is a complex process of sequential phases that begins with an inflammatory response and hematoma caused by tissue reactions on vascular and soft tissue injury [2]. Nonunion or similar problems occur during healing in 5–10% of all fractures [3]. Despite the lack of a definitive consensus, nonunion is clinically characterized by motion between the fracture parts and a persistent fracture line on the radiograph. This eventually results in the development of synovial pseudoarthrosis six to nine months after the initial trauma [4]. In general, risk factors for nonunion are classified either as fracture-related or patient-related factors. One of the patient-related risk factors is nutrition and amino acids and minerals in particular [5]. Arginine is a semi-essential amino acid with well-established roles in growth, tissue repair, immune regulation, and vascular homeostasis. Experimental and clinical evidence indicates that arginine is required for normal spermatogenesis, embryonic and fetal development, neonatal growth, and the regulation of vascular tone and hemodynamics. In addition, arginine supplementation has been shown to accelerate wound healing, enhance insulin sensitivity, and support collagen synthesis through its involvement in growth hormone, polyamine, and proline metabolism. Dietary arginine also serves as a critical substrate for protein synthesis, cell proliferation, T-lymphocyte function, and maintenance of positive nitrogen balance [6]. The bioavailability and biological efficacy of arginine can be enhanced when administered as a complex with inositol and silicon [7, 8]. In this context, previous experimental studies have demonstrated that oral supplementation with the arginine–silicate–inositol (ASI) complex significantly improves bone mineral density and mineral composition (Ca, Mg, P, Mn) in avian models [8]. Moreover, ASI supplementation has been reported to enhance bone regeneration in rat calvarial defect models, highlighting its potential role in supporting bone repair processes [9].

The arginine-silicate-inositol complex (ASI; arginine 49.47%, silicon 8.2%, and inositol 25%) represents a novel and bioavailable source of arginine and silicon with demonstrated safety and potential benefits for vascular and skeletal health [10, 11]. Despite accumulating evidence supporting the positive effects of ASI on bone metabolism and defect healing, its impact on fracture healing and bone union has not been directly investigated. Therefore, the aim of the present study was to evaluate the effects of oral ASI supplementation on femoral fracture healing in a rat model, with a specific focus on radiological and histopathological indicators of callus formation, osteoblast and osteoclast activity, and fracture consolidation.

## Materials and Methods

### Animals and Study Design

The sample size calculation indicated the need for a total of 28 animal subjects (F test, a priori, effect size 0.75, 85% power, α-error = 0.05. A total of 28 female Wistar-Albino rats, 8 weeks old, weighing 200 to 250 g were used in the study. Throughout the study, the rats were maintained under controlled conditions for temperature (23 ± 2 °C), humidity (55 ± 10%) and automated lighting (a 12:12-h light-dark cycle. Young adult rats were selected because femoral diaphyseal osteotomy models with intramedullary fixation in 8-12-week-old rodents are widely validated for intervention testing, providing reproducible and well-characterized fracture-healing kinetics that allow sensitive detection of treatment-related differences in callus formation and consolidation [12–15]. Animals were randomly allocated to the experimental groups using a simple randomization method, as shown in Table 1. A non-fracture control group (Group 1) was included to establish baseline radiological and histopathological characteristics of intact femoral bone, enabling differentiation between physiological bone remodeling and fracture- or treatment-induced changes. This study was approved by the university’s ethics committee on animal experiments (Approval No:2019/55).

**Table 1:** Study groups

Groups	Treatments
Group 1	Control (without fracture induction-standard diet)
Group 2	Control (with fracture induction-standard diet)
Group 3	With fracture induction-low dose ASI
Group 4	With fracture induction-high dose ASI

The ASI doses for the study were determined according to the literature [16, 17]. ASI obtained from a commercial company (Nutrition 21, LLC, NY, America), was prepared at low (4.14 mg/rat/day) and high (8.28 mg/rat/day) doses, mixed with drinking water. The ASI doses were administered by oral gavage under direct observation to ensure accurate and complete dose delivery. Each animal was individually monitored during and immediately after administration to confirm that the full intended dose was delivered without regurgitation, aspiration, or spillage. Animals showing any signs of incomplete administration would have been excluded; however, no incomplete dose administrations were observed during the study period. The selected ASI doses were based on prior preclinical studies demonstrating efficacy and safety. In particular, Demir et al. [18], reported significant biological effects using ASI at 4.14 mg/rat/day administered orally in 8-week-old rats, without evidence of toxicity. Accordingly, this dose was selected as the low-dose ASI, while 8.28 mg/rat/day was included to evaluate potential dose-dependent effects. These doses are also consistent with the pharmacological range derived from human studies using approximately 1.500–1.600 mg/day ASI, taking into account interspecies dose-scaling considerations [19, 20].

## Surgical Procedure

Anesthesia was achieved by intramuscular injection of xylazine (10 mg/kg) and ketamine (100 mg/kg) to all animals undergoing surgical procedures. After anesthesia, the rats were placed in the lateral decubitus position on the operating table, with the right femur on top. The right femur and knee were cleaned with 10% iodine before and after the operation. A 3-cm longitudinal skin incision was made lateral to the right femur. The femur was accessed through the intermuscular plane. Osteotomy was performed on the middle 1/3 part of the femur using the multi-drilling technique. Then, a Kirschner wire was introduced for intramedullary fixation.

**Fig. 1:**
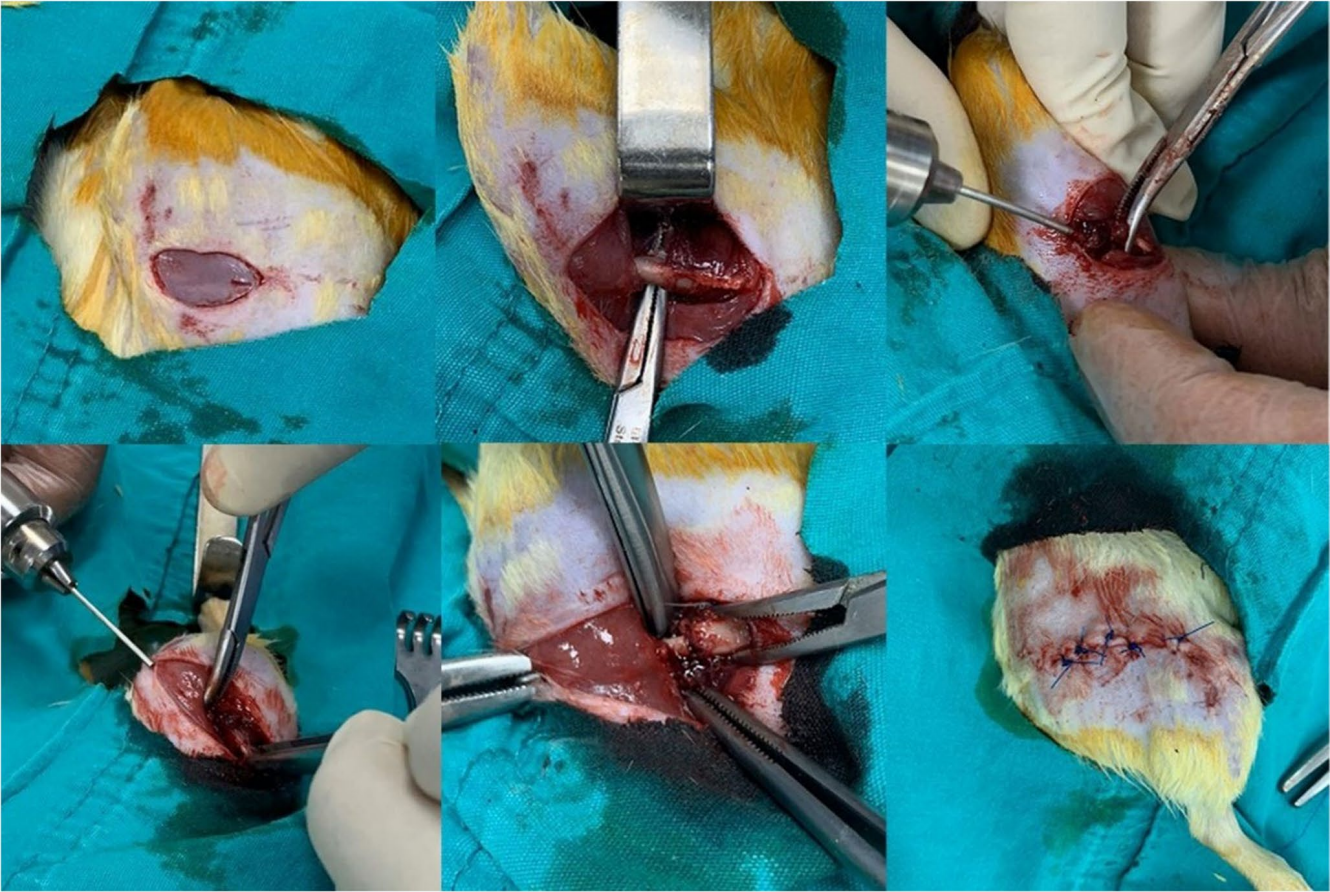
Induction of open femur fracture and intramedullary pin fixation using multiple puncture techniques

The incision was closed with a 4.0 polyglactin absorbable suture. Antibiotic prophylaxis (cefazolin) was given before and after surgery. For postoperative pain management, buprenorphine 0.05 mg/kg was administered twice a day. The animals were permitted full weight-bearing after the surgery. Groups 1 and 2 were fed a standard diet, Group 3 low-dose ASI, and Group 4 high-dose ASI for 8 weeks. After the treatment period, bidirectional radiographs were acquired from the right femur of the rats. Then, the animals were sacrificed by non-anesthetic decapitation. The right femur was removed with hip and knee disarticulation. The removed femoral area was freed of all soft tissues. The Kirschner wires in the medulla of the femur were removed. The bones were sent for histopathological examination in 10% buffered formaldehyde solution (Fig. 1).

## Radiological Assessment

After the treatment period, bidirectional radiographs (AP and lateral) were acquired from the right femur of the rats. Acquisitions were performed using an X-Ray device (Siemens Healthcare, Erlangen, Germany) at 45 cm and 30 kW-15–20 s. A radiologist evaluated results. Radiological evaluations were performed by an experienced radiologist who was blinded to the group assignments. Union was determined by visually evaluating the mineralized callus bridging in the fracture line on AP and lateral radiographs (bridging on the right side, 1 point, and on the left side, 1 point, on both AP and lateral radiographs [21].

## Histopathological Assessment

Bone specimens were demineralized by soaking in 10% formic acid solution for 72 h. The specimens were then dehydrated and embedded in paraffin. Six-µm sections were obtained at the fracture level using a microtome and stained with hematoxylin and eosin. The stained preparations were examined under a light microscope (BX51, Olympus, Tokyo, Japan) and visualized using a digital camera (Olympus Corporation). For histomorphometric analysis, osteoblast and osteoclast quantification was performed using a static histomorphometric approach. Cell counts were obtained from systematically and randomly selected microscopic fields within a predefined region of interest encompassing the fracture callus. For each section, multiple non-overlapping high-power fields (×400 magnification) were analyzed, and mean values were calculated for each animal to reduce sampling variability, consistent with established histomorphometric recommendations [22, 23]. Histopathological evaluations were conducted by an investigator blinded to the experimental groups. Osteoblast and osteoclast identification and scoring were performed according to the criteria and scoring system described by Huo et al. [24]. Use of a single blinded observer and predefined morphological criteria is a recognized approach to minimize inter-observer variability in manual bone histomorphometry [25, 26]. In addition, lamellar bone formation was assessed and scored as an indicator of new bone formation (Table 2).

**Table 2:** Osteoblast and bone density

Osteoblast Density	Bone Formation Density
0 No osteoblast	0 No bone formation
1 Mild osteoblast	1 Mild bone formation
2 Dense osteoblasts	2 Moderate bone formation
	3 Marked bone formation

### Statistical Analysis

Data analyses were performed using SPSS software (Statistical Package for Social Sciences; SPSS Inc., Chicago, IL, USA) version 22. Descriptive data were expressed as number and percentage (n, %) for categorical and ordinal variables, and as mean ± standard deviation (SD) for semi-continuous outcomes. Differences in score distributions among groups were analyzed using Pearson’s Chi-square test. In addition, because the experimental groups followed a biologically ordered, dose-dependent structure (control → fracture control → low-dose ASI → high-dose ASI), the Linear-by-Linear Association test was employed to assess trend effects across groups. In addition, radiological fracture-healing scores were analyzed using one-way analysis of variance (ANOVA), followed by Tukey’s post hoc test for multiple comparisons. A p-value of < 0.05 was considered statistically significant. The non-fracture control group (Group 1) was included in the analyses to provide baseline reference values for normal bone histology and radiological appearance, enabling fracture and treatment-related changes to be interpreted relative to physiological bone remodeling.

## Results

### Histopathological Results

**Fig. 2:**
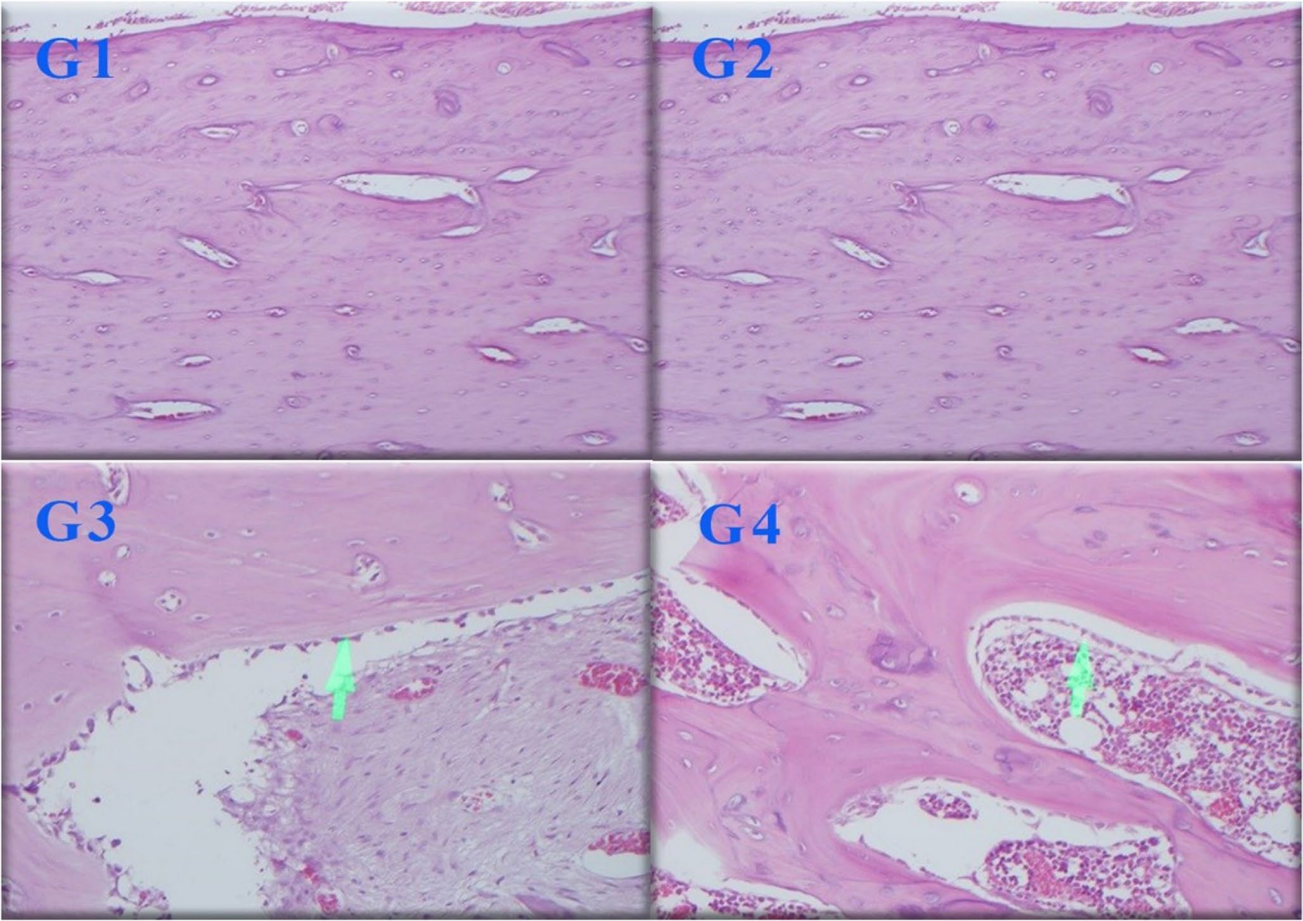
Histomorphometric assessment of the effect of ASI on fracture healing. While no osteoblast formation was detected in Group 1, the highest osteoblast formation was present in Group 4 (*p* < 0.001). Increased osteoblast activity in high-dose ASI groups (Group 3 and Group 4) (arrows) Osteoclast formation was significantly decreased in Group 4 (*p* = 0.017) (Hematoxylin and eosin, 40X)

Osteoblast formation in the groups was evaluated histopathologically. While no osteoblast formation was detected in Group 1, the highest osteoblast formation was present in Group 4 (Table 3) (*p* < 0.001). Osteoclast formation was significantly decreased in Group 4 (*p* = 0.017) (Fig. 2).

**Table 3:** The effect of ASI on histopathological changes

	Osteoblasts			Osteoclasts	
	No osteoblast	Mild osteoblasts	Dense osteoblasts	No bone formation	Mild bone formation
	*n* (%)	*n* (%)	*n* (%)	*n* (%)	*n* (%)
Group 1	6 (85.7)	1 (14.3)	0 (0.0)	7 (100.0)	0 (0.0)
Group 2	0 (0.0)	5 (71.4)	2 (28.6)	7 (100.0)	0 (0.0)
Group 3	0 (0.0)	3 (42.9)	4 (57.1)	5 (71.4)	2 (28.6)
Group 4	0 (0.0)	1 (14.3)	6 (85.7)	4 (57.1)	3 (42.9)
p-value *X*^2^-value	*p* < 0.0001 *X*^2^*=*29.067			*p =* 0.0171 *X*^2^*=*5.682	

Data are presented as number of animals (n) and percentage (%). Histopathological scores for osteoblast density and bone formation were evaluated using ordinal scoring systems. Group comparisons were performed using Pearson’s Chi-square test. Because the experimental groups followed a biologically ordered, dose-dependent structure, the Linear-by-Linear Association test was additionally applied to assess trend effects across groups. Statistical significance was accepted at *p* < 0.05

## Radiological Results

**Fig. 3:**
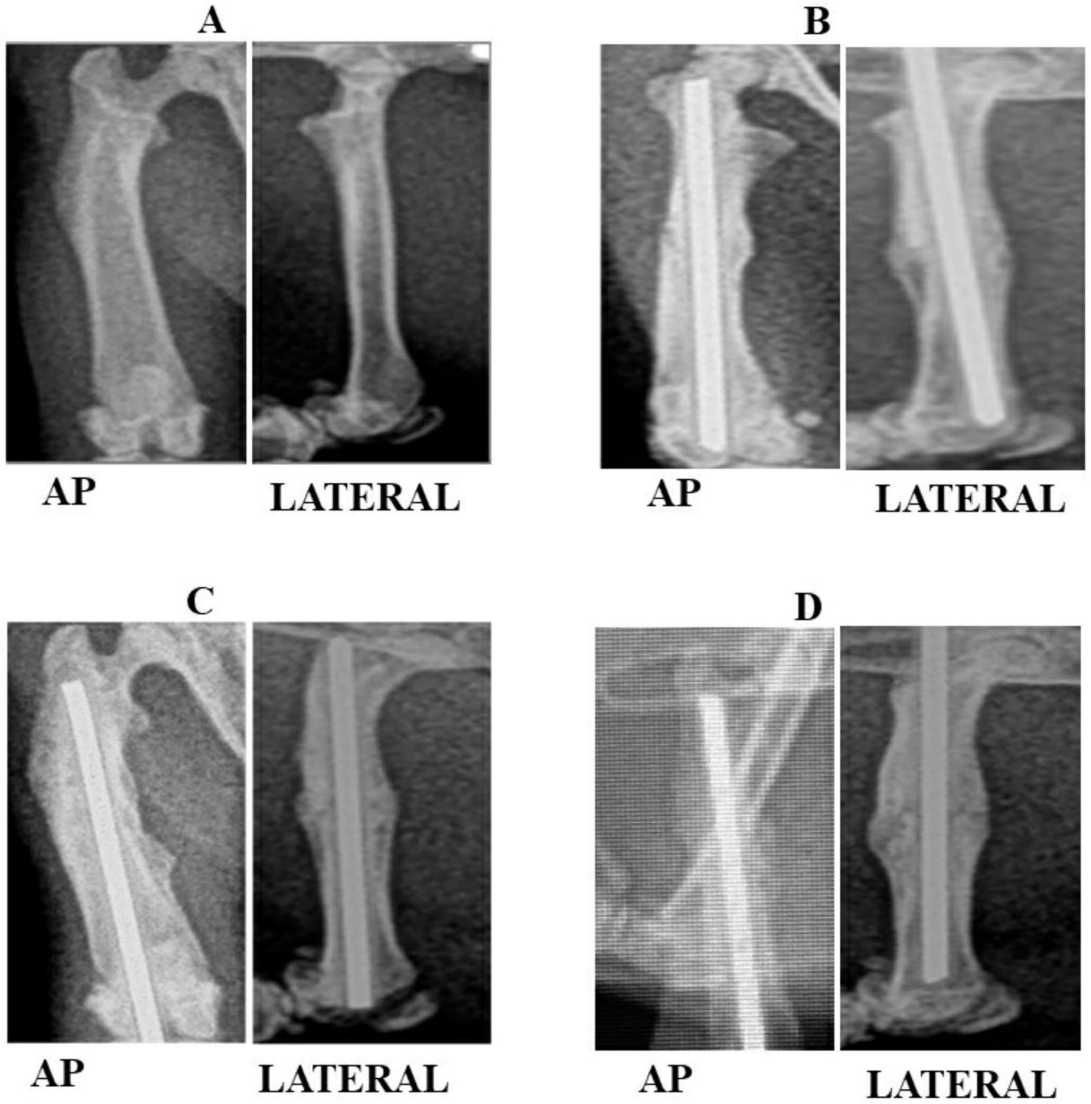
Radiographic assessment of the effect of ASI on fracture healing. The fracture line was still prominent in the control group (Group 2) with fracture induction, and no union was achieved. While union with normal callus tissue was observed in the low-dose ASI group (Group 3), the high-dose ASI group (Group 4) had union with large and mature callus tissue.**A**: Control group (without fracture induction-standard diet), **B**: Control group (with fracture induction-standard diet), **C**: With fracture induction-low-dose ASI (Group 3), **D**: With fracture induction-high dose ASI (Group 4)

**Fig. 4:**
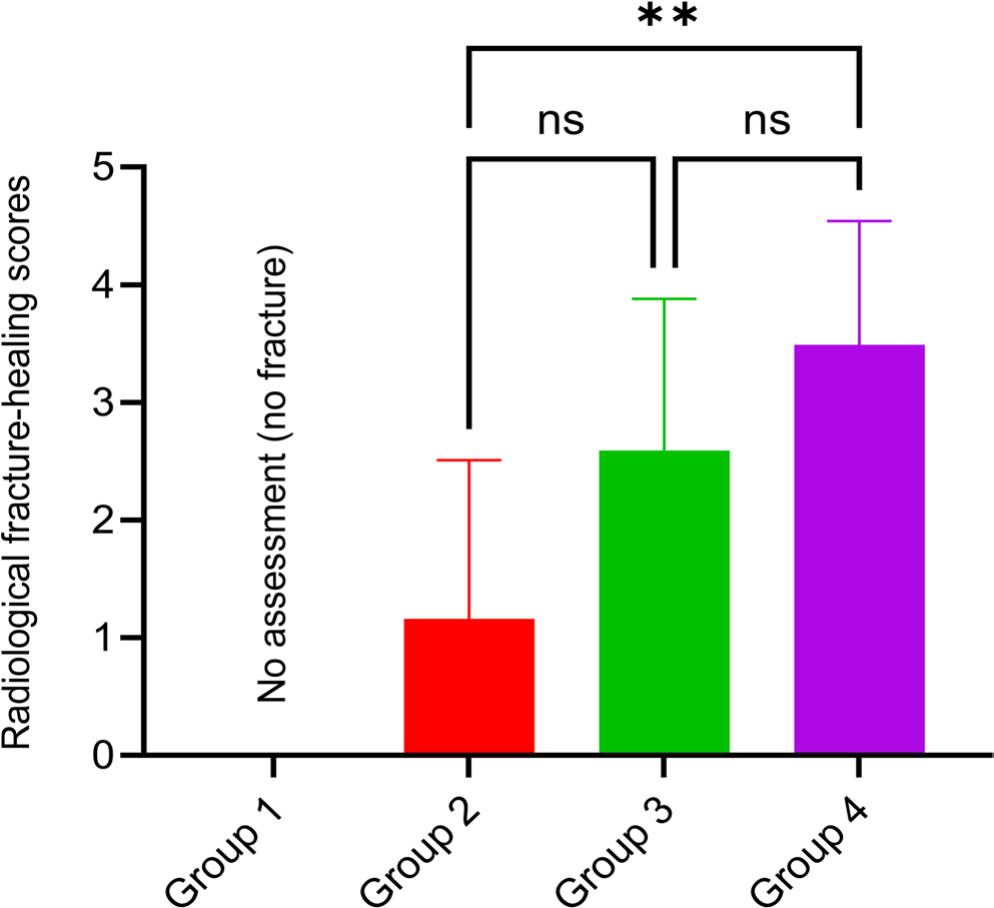
Radiological fracture-healing scores in experimental groups. Group 1 was not assessed radiologically due to the absence of fracture. Group 2 (fracture control) exhibited low scores, indicating lack of union. Group 3 (low-dose ASI; 4.14 mg/rat/day) showed increased scores consistent with callus formation, while Group 4 (high-dose ASI; 8.28 mg/rat/day) demonstrated the highest scores, reflecting more mature and extensive callus formation. Data are presented as mean ± SD. **p < 0.01 indicates a significant difference between the indicated groups; ns, not significant

The AP and lateral femoral radiographs acquired after eight weeks are presented in Fig. 3. The results of the assessment of these radiographs by an expert radiologist are summarized in Table 4. According to these radiological results, the fracture line was still prominent in the control group (Group 2) with fracture induction, and no union was achieved. While union with normal callus tissue was observed in the low-dose ASI group (Group 3), the high-dose ASI group (Group 4) had union with large and mature callus tissue (Fig. 3). These differences between the groups were statistically significant. Radiological evaluation demonstrated clear differences in fracture healing among the experimental groups. As expected, Group 1 (non-fracture control) was not subjected to radiological scoring. In Group 2 (fracture control), the eight-week radiographic score remained low, indicating absence of effective fracture union and persistence of a visible fracture line (Fig. 4). In contrast, ASI-treated groups exhibited improved radiological outcomes. Group 3 (low-dose ASI) showed higher eight-week scores compared with the fracture control group, reflecting partial fracture union with callus formation; however, this difference did not reach statistical significance (ns). Notably, Group 4 (high-dose ASI) demonstrated the highest radiological scores, consistent with more extensive and mature callus formation and advanced fracture consolidation. The overall comparison revealed a statistically significant improvement in fracture healing in the high-dose ASI group compared with the fracture control group (*p* < 0.01). No significant difference was observed between the low- and high-dose ASI groups (ns), indicating a trend toward dose-dependent improvement.

**Table 4:** Radiographic assessment

	No of Subjects	Eight-Week Score (Mean ± SD)	*p*-value
Group 1	7	No assessment (no fracture)	
Group 2	7	1.16 ± 1.35	0.0083
Group 3	7	2.59 ± 1.29	
Group 4	7	3.49 ± 1.05	
*Multiple comparisons*			
	Group 2 vs. Group 3		0.1052
	Group 2 vs. Group 4		0.0065
	Group 3 vs. Group 4		0.3812

Radiological fracture healing was assessed eight weeks after fracture induction using an ordinal scoring system based on anteroposterior and lateral radiographs. Data are presented as mean ± standard deviation (SD). Group 1 was not included in the radiological scoring because no fracture was induced. Overall group differences were evaluated using appropriate non-parametric statistical tests for ordinal data, followed by pairwise post hoc comparisons. Reported p-values indicate between-group comparisons as specified. Statistical significance was defined as *p* < 0.05

## Discussion

The biology of fracture healing is a complex process that follows specific regenerative patterns and involves coordinated cellular and tissue-level changes across inflammatory, reparative, and remodeling phases. There is much to be learned to comprehend the mechanisms of bone regeneration fully. The biological process includes an inflammatory phase that involves the production and release of several important molecules and the recruitment of mesenchymal stem cells to form a primary cartilage callus. This primary callus then undergoes revascularization and calcification and is finally remodeled to restore a normal bone structure fully. The bone healing process involves a variety of cellular components that are essential for the progress of healing. Inflammatory cells (T-cells, B-cells, mast cells, macrophages, eosinophils, and neutrophils) are the first cellular components of the fracture environment. This is followed by the mesenchymal progenitor cells, endothelial cells, chondrocytes, osteoblasts, and finally, osteoclasts [27]. While osteoblasts and mature osteocytes set a solid ground for obtaining and maintaining normal bone mass, osteoclasts are considered the only cell type responsible for bone resorption. These cells, which are the most important cells during osteogenesis and remodeling, do not act independently from each other. Therefore, these two important cells play a vital role in fracture healing. During bone healing, these cells are expected to increase at the fracture site [28]. Several studies have been conducted on the factors affecting the healing of fractures [29–32]. Many studies have drawn attention to the similarity, in several aspects, between fracture healing and the growth and development of a bone. Factors such as osteoblasts, osteoclasts, vascular cells, inflammatory cells and proinflammatory cytokines, play important roles in the healing of a fracture just like the development of a bone [2, 33]. Direct differentiation of mesenchymal progenitors to osteoblasts during bone union, intramembranous ossification, is a specific mechanism of bone repair in stable fractures, but also occurs along the periosteal and endosteal surfaces of the bone in less stable fractures. Osteoclasts are myeloid-origin multinucleated cells that form tight connections to the bone surface through a special membrane structure called the sealing zone. Osteoclast activity is essential to fracture healing, induced near the end of healing, and it reshapes the hard callus and restores the bone to dimensions similar to those before the injury [27]. Recent studies show that arginine and silicon play key roles in the development, growth, and remodeling of long bones [34]. Arginine is effective in calcium absorption, the calcification of bones and teeth, the treatment of rickets and osteomalacia, osteogenesis, and the synthesis of extracellular matrix in bone and cartilage tissues [35–37]. Silicon, in turn, plays a role in ossification together with calcium in collagen synthesis and the synthesis of the protein matrix in the cartilage [38, 39]. Arginine participates in the synthesis of substrates involved in collagen production and is associated with increased levels of growth hormone, insulin-like growth factor (IGF)-1, and nitric oxide, which are known to support bone matrix formation [40]. Arginine is known to substantially increase certain parameters called bone formation markers, such as alkaline phosphatase, nitric oxide, type I collagen, and IGF. These markers act on bone tissue at the cellular level, increasing the synthesis of the bone matrix. In addition, it has been shown that the effects of arginine on bone tissue are directly associated with its effect on the cell proliferation mechanism [41]. Silicon is considered an important component in bone formation, and its deficiency has a negative impact on skeletal development. ASI, composed of arginine, silicate and inositol, shows greater bioavailability than its components due to its physicochemical properties. ASI is considered a promising agent because of these properties. The literature offers limited research on osteoblastic metabolism, connective tissue and vascular collagen metabolism, and the ASI complex in osteoporosis. Nevertheless, the positive immunological activities of arginine and silicon are well known and both have been identified to affect the osteoblastic activity and wound healing in bones and connective tissues [42]. Considering all this information, the present study was conducted with the notion that ASI could have positive effects on the healing of fractures, as it has in the case of bone development. Sahin et al. [8] examined the effects of ASI on bone tissue in a study on poultry fed a diet supplemented with 500 mg/kg and 1.000 mg/kg. The authors established increases in bone mineral density, calcium and phosphorus, serum levels of osteocalcin, and alkaline phosphatase in both experimental groups, depending on the dose compared to controls. In our study, bone formation and bone metabolism associated with bone remodeling and bone tissue healing were statistically significantly higher in the ASI-treated group compared to the controls (e.g., the numbers of osteoblasts and osteoclasts and the ​​new bone formation site). Osteoblasts and osteoclasts act together in the healing and remodeling of bone tissue. This study observed a significant increase in these cell groups in the ASI-treated groups. In addition, the radiological results revealed better outcomes in the ASI-treated groups. Although the present study demonstrates that ASI supplementation enhances callus formation and fracture consolidation, the translational scope of the experimental model should be interpreted with caution. The use of young adult rats provides a controlled and reproducible platform to evaluate treatment-related effects on the intrinsic biology of fracture healing and is widely adopted in femoral and tibial osteotomy models employing intramedullary fixation [12–15]. In contrast, aged and estrogen-deficient models are primarily designed to investigate delayed or impaired healing associated with osteoporosis and inflammaging and introduce additional systemic factors that may obscure the direct bone response to a specific intervention [43–46]. Therefore, while the current findings support a direct pro-healing effect of ASI under biologically stable conditions, further studies using ovariectomized and/or aged fracture models will be important to define the relevance of ASI supplementation in osteoporotic and geriatric fracture settings [47, 48].

An interesting finding of the present study was the reduction in osteoclast numbers observed in the high-dose ASI group. Osteoclasts play a central role in bone remodeling and late-stage fracture healing, not only through resorption but also by providing critical coupling signals that regulate osteoblast recruitment and activity [49–51]. Dysregulation of osteoclast activity-either excessive or insufficient-has been associated with impaired remodeling and delayed fracture maturation [45, 52]. The decrease in osteoclast numbers at higher ASI doses may reflect a dose-dependent antiresorptive effect, potentially attributable to the silicon component of ASI, which has been shown to inhibit osteoclastogenesis and bone resorption in experimental models [53, 54]. Such an effect could be advantageous by limiting excessive resorption during early consolidation, thereby supporting callus stability and net bone formation. However, excessive or prolonged suppression of osteoclast activity may theoretically slow late-stage remodeling, as observed with potent antiresorptive therapies [52, 55]. Accordingly, the osteoclast findings in the high-dose ASI group highlight the importance of dose and temporal dynamics in ASI-mediated fracture repair and warrant further investigation using time-resolved analyses to delineate early versus late remodeling effects.

In this study, it was observed that radiological and histopathological results were compatible with each other. The radiological assessment showed that the fracture line was still prominent in the non-ASI-treated group (Group 2) and no union was achieved. On the other hand, the ASI-treated groups (Group 3 and Group 4) achieved better radiological results, with the fracture union with callus tissue and osteoblast density. We further established that a higher number of and more mature fracture healing tissue developed in the high-dose ASI group compared to the low-dose ASI group. This result showed a dose-dependent positive effect of ASI on fracture healing. The finding of the histopathological assessment revealing a higher number of both osteoblasts and osteoclasts in the high-dose ASI group (Group 4) showed histologically that the positive effect of ASI on union could increase according to the dose.

Previous studies indicate that oral gavage can induce mild to moderate, often transient stress responses in rodents, reflected by increases in corticosterone levels, heart rate, or anxiety-like behaviors [56–60]. Repeated gavage has also been reported to activate the hypothalamic–pituitary-adrenal axis in certain strains, particularly when animals are not habituated to handling [57, 61–63]. Importantly, the literature also demonstrates that gavage-related stress can be substantially reduced when procedures are performed by experienced personnel and animals are acclimatized to handling, with stress biomarkers and health parameters remaining comparable to those of controls [58, 64, 65]. In the present study, all experimental groups were subjected to identical handling and oral gavage procedures, thereby balancing any potential stress-related effects across groups and minimizing systematic bias. Moreover, the consistent, dose-dependent improvements in radiological and histopathological outcomes in ASI-treated animals argue against a nonspecific stress-related explanation and instead support a genuine biological effect of ASI on fracture healing. Nevertheless, in line with recent methodological refinements, future studies may benefit from employing voluntary oral administration strategies such as micropipette-guided drug administration, palatable gels, or diet-based delivery, which have been shown to preserve bioavailability while significantly reducing stress-related endocrine and behavioral responses compared with conventional gavage [57, 59, 60, 66–69]. Adoption of such approaches would further strengthen translational interpretation by minimizing residual confounding effects associated with repeated oral gavage.

From a clinical perspective, fracture healing is primarily optimized through mechanical stabilization, appropriate load sharing, and, in selected cases, adjunctive biophysical or pharmacological interventions. Established strategies such as stable fixation with controlled micromotion, electromagnetic or electrical stimulation, and selected biological agents, including parathyroid hormone (PTH) analogues or bone morphogenetic proteins (BMPs) have demonstrated efficacy in accelerating fracture repair under specific clinical conditions, particularly in delayed union or compromised healing scenarios [70–72]. Compared with these interventions, the present findings position ASI as a nutritional-biological support strategy rather than a primary fracture-healing therapy. The observed enhancement of callus formation, osteoblastic activity, and fracture consolidation with ASI supplementation aligns with previous experimental evidence showing that arginine and silicon contribute to bone mineralization, collagen synthesis, and osteogenic signaling [9, 73, 74]. In this context, ASI appears conceptually closer to other bone-supportive nutritional or metabolic interventions that strengthen the biological substrate of repair, rather than to potent anabolic or antiresorptive drugs that directly modulate fracture healing kinetics. Importantly, while agents such as PTH or biophysical stimulation have demonstrated the ability to shorten healing time in selected clinical settings, comparable clinical evidence for ASI in human fracture healing is currently lacking.

Several limitations of the present study should be acknowledged. Fracture healing was evaluated in young adult female rats, a model selected to provide experimental control and reproducibility when assessing the direct biological effects of ASI on bone repair. This approach is widely accepted and routinely applied in standardized femoral and tibial osteotomy models for intervention testing [12–15]. However, it does not fully recapitulate the clinical complexity of fracture healing in aging or osteoporotic conditions. Both advanced age and estrogen deficiency have been shown to impair angiogenesis, inflammatory resolution, and mineral accretion during fracture repair, resulting in delayed or incomplete healing [43–46]. In addition, fracture healing was evaluated in young adult female rats under controlled conditions, which may not fully recapitulate the complex clinical setting of age-related or osteoporotic fractures. In addition, the relatively limited sample size due to ethical considerations and the reliance on two-dimensional radiography and histopathological assessment **should be noted.** The lack of advanced three-dimensional imaging techniques, such as micro-computed tomography (micro-CT), precluded detailed assessment of callus microarchitecture, trabecular organization, and volumetric bone parameters. Incorporating micro-CT, along with biomechanical testing and biochemical markers, into future studies would enable a more comprehensive, quantitative evaluation of fracture healing and further strengthen translational relevance. Finally, the present study focused primarily on morphological and cellular outcomes and did not include molecular or immunohistochemical analyses. Assessment of key osteogenic, angiogenic, and osteoclast-regulatory markers, including Runx2, Osterix, VEGF, TRAP, and the RANKL/OPG axis, would provide deeper mechanistic insight into the pathways through which ASI modulates fracture healing [49, 50, 70]. Future studies integrating molecular profiling with advanced imaging and biomechanical testing, particularly in aged and osteoporotic fracture models, will be essential to strengthen the translational relevance of ASI as a supportive strategy for bone repair.

## Conclusion

This study demonstrates that oral supplementation with the arginine–silicate–inositol (ASI) complex enhances femoral fracture healing in rats. Radiological and histopathological findings revealed improved callus formation, fracture union, and new bone formation in ASI-treated groups, with more pronounced effects at the higher dose, indicating a dose-dependent response. While these findings support a bone-healing-promoting effect of ASI under controlled conditions, further studies using advanced imaging, biomechanical testing, and clinically relevant fracture models are required to define its translational potential.

## Data Availability

The data that support the findings of this study are available from the corresponding author upon reasonable request.
